# Lateral parabrachial nucleus astrocytes control food intake

**DOI:** 10.3389/fendo.2024.1389589

**Published:** 2024-06-03

**Authors:** Devesh Mishra, Jennifer E. Richard, Ivana Maric, Olesya T. Shevchouk, Stina Börchers, Kim Eerola, Jean-Philippe Krieger, Karolina P. Skibicka

**Affiliations:** ^1^ Department of Physiology/Metabolic Physiology, Institute of Neuroscience and Physiology, The Sahlgrenska Academy at the University of Gothenburg, Gothenburg, Sweden; ^2^ Campbell Family Mental Health Research Institute, Centre for Addiction and Mental Health (CAMH), Toronto, ON, Canada; ^3^ Department of Nutritional Sciences, Pennsylvania State University, University Park, PA, United States; ^4^ Research Centre for Integrative Physiology and Pharmacology, Institute of Biomedicine, University of Turku, Turku, Finland; ^5^ Institute of Veterinary Pharmacology and Toxicology, University of Zurich - VetSuisse, Zurich, Switzerland; ^6^ Huck Institutes of the Life Sciences, Pennsylvania State University, University Park, PA, United States

**Keywords:** lateral parabrachial nucleus, GLP-1, ghrelin, hindbrain, astrocytes, glia, NMDA, high-fat diet

## Abstract

Food intake behavior is under the tight control of the central nervous system. Most studies to date focus on the contribution of neurons to this behavior. However, although previously overlooked, astrocytes have recently been implicated to play a key role in feeding control. Most of the recent literature has focused on astrocytic contribution in the hypothalamus or the dorsal vagal complex. The contribution of astrocytes located in the lateral parabrachial nucleus (lPBN) to feeding behavior control remains poorly understood. Thus, here, we first investigated whether activation of lPBN astrocytes affects feeding behavior in male and female rats using chemogenetic activation. Astrocytic activation in the lPBN led to profound anorexia in both sexes, under both *ad-libitum* feeding schedule and after a fasting challenge. Astrocytes have a key contribution to glutamate homeostasis and can themselves release glutamate. Moreover, lPBN glutamate signaling is a key contributor to potent anorexia, which can be induced by lPBN activation. Thus, here, we determined whether glutamate signaling is necessary for lPBN astrocyte activation-induced anorexia, and found that pharmacological N-methyl D-aspartate (NMDA) receptor blockade attenuated the food intake reduction resulting from lPBN astrocyte activation. Since astrocytes have been shown to contribute to feeding control by modulating the feeding effect of peripheral feeding signals, we further investigated whether lPBN astrocyte activation is capable of modulating the anorexic effect of the gut/brain hormone, glucagon like peptide -1, as well as the orexigenic effect of the stomach hormone - ghrelin, and found that the feeding effect of both signals is modulated by lPBN astrocytic activation. Lastly, we found that lPBN astrocyte activation-induced anorexia is affected by a diet-induced obesity challenge, in a sex-divergent manner. Collectively, current findings uncover a novel role for lPBN astrocytes in feeding behavior control.

## Introduction

Global obesity rates continue to grow unabated. Therefore, there is a tremendous interest in developing new more effective pharmacological interventions. Food intake is controlled by the central nervous system (CNS). This control has been primarily ascribed to the hypothalamus, yet, in recent decades, a wealth of literature has indicated that many other brain areas are crucial to food intake control, including multiple nuclei in the hindbrain. The view on which CNS cell types are contributing to food intake controls has also been similarly expanded in recent years to include not only neurons but also astrocytes (1–3). Astrocytes represent a diverse group of cells that vary based on appearance, function, and distribution. Astrocytes control the concentrations of ions, neurotransmitters, and neurohormones in the extracellular space: they can release neurotransmitters (for example, glutamate) and other signaling molecules (for example, ATP, adenosine, or prostaglandins) as well as supply neurons with neurotransmitter precursors (4). This allows astrocytes to modulate neuronal activity and synaptic transmission.

Recent preclinical studies suggest a potential role for astrocytes in food intake control (1–3). Activation of astrocytes in the hypothalamus attenuates the hunger hormone (ghrelin)-mediated orexigenic behavior, while satiety hormone (leptin) anorexigenic effects can be potentiated by astrocytic activation (1, 2, 5). Moreover, astrocytes are capable of directly sensing energy status, as they express receptors for ghrelin, leptin, insulin, and glucose (5–9). One of the mechanisms via which astrocytes can modify energy intake involves glutamatergic signaling; in fact, glutamate can be released by astrocytes upon stimulation (10, 11), and this astrocytic glutamate release is capable of modifying neuronal activity (12, 13).

To date, most investigations of astrocytic contribution to food intake control focused on the hypothalamus, while a few focus on the nucleus of the solitary tract (NTS) of the hindbrain (14–16). However, another hindbrain region—the lateral parabrachial nucleus (lPBN)—is well-positioned to sense energy requirement signals and mediate satiety signals culminating in meal termination (17–22). The potential influence of this brain area on feeding behavior is so robust that disinhibition of the lPBN leads to severe anorexia in mice (23). There is also evidence suggesting that this potent feeding inhibition requires glutamate signaling within the lPBN as severe anorexia can be rescued pharmacologically by blocking the glutamate-specific N-methyl-D-aspartate NMDA-NR2B subunits (23). However, whether astrocytes in this brain area contribute to feeding behavior control is poorly understood, including the potential role of glutamatergic signaling in this process.

This study aimed to investigate whether activation of lPBN astrocytes affects feeding behavior in male and female rats. To achieve this, we utilized chemogenetic activation of lPBN astrocytes by astrocyte-specific excitatory designer receptors exclusively activated by designer drugs (DREADDs). More specifically, excitatory AAV-hm3dq was applied to the lPBN of male and female rats. Astrocytic contribution to food intake control was tested during the dark cycle when rats normally consume most of their calories and also after a food deprivation challenge. Since astrocytes have an important contribution to glutamate homeostasis, and can themselves release glutamate, and lPBN glutamate signaling is a key contributor to potent anorexia which can be induced by lPBN activation, we determined whether glutamate signaling at the NMDA receptor is necessary for lPBN astrocyte activation-induced anorexia using pharmacological NMDA receptor blockade. Considering that previous literature has indicated that astrocytes contribute to feeding control by modulating the feeding effect of peripheral feeding signals, we further investigated whether lPBN astrocyte activation can modulate the anorexic effect of the gut/brain hormone, glucagon like peptide -1, as well as the orexigenic effect of the stomach hormone - ghrelin. Lastly, we determined whether lPBN astrocyte-induced anorexia is affected by a diet-induced obesity challenge.

## Materials and methods

### Animals

Male and female Sprague–Dawley rats (5 weeks at arrival, Charles River, Germany) were individually housed in cages with *ad-libitum* access to chow or exposed to a high-fat and sucrose diet (HFSD; *ad-libitum* access to chow, in-house made 50%/50% by weight sucrose/lard mixture) or a high-fat high-sucrose (HFHS) consisting of *ad-libitum* access to chow, in addition to 30% sucrose solution and lard separately. This diet was used to enable measurements of preference for each macronutrient. All rodents had *ad-libitum* access to water and were maintained on a 12-h/12-h light/dark cycle. All studies were approved by the Animal Welfare Committee of the University of Gothenburg, Sweden, Ethical permit # 137/15 and 77/15.

### Drugs

Clozapine-N-oxide (CNO) was purchased from Larodan AB, Stockholm, and dissolved in 0.5%–1% DMSO (St. Louis, MO, United States: Sigma-Aldrich). For central injections, all the other drugs were dissolved in artificial cerebrospinal fluid (aCSF; Tocris, United Kingdom). The GLP-1 receptor (GLP-1R) agonist exendin-4 (Ex-4), ghrelin, and the NMDA receptor blocker Ro25–6981 were purchased from Tocris, United Kingdom. For systemic injections, CNO was dissolved in saline.

### Intracranial cannulation and viral infusion

A bilateral guide cannula targeting the rat lPBN was implanted under ketamine and xylazine anesthesia. The coordinates were 2.0/−9.2/4.5 mm (midline/bregma/skull, respectively) (20) with the injector aimed 6.5 mm ventral to the skull. We have previously successfully targeted the lPBN using this approach (20, 22, 24). LPBN-targeted bilateral infusion of AAV5/GFAP-hm3D(Gq)-mCherry (addgene viral prep 50478-AAV5) or rAAV5/GFAP-HA-hm3D-IRES-mCitrine (UNC vector core) was performed to express DREADDs on lPBN astrocytes or AAV5/GFAP-mCherry for DREADD-negative controls at 0.4 µl volume and at the rate of 0.1 µl/min. The viral titers used ranged from 4 × 10^12^ to 7 × 10^12^ (vg/ml). The injector (33 gauge) was left for 10 min after injections to allow for proper diffusion. Experiments began 3 weeks following viral injections. Cannula placements and AAV expression were confirmed postmortem using 20 µM of brain sections stained with DAPI and observed under a confocal microscope. The viral vector used was confirmed to be astrocyte-specific by many others (10, 16, 25–27), where it was reported that over 99% of cells targeted by AAV using the GFAP promoter were astrocytes, conferring a very high degree of specificity.

### Acute food intake measurement experiments

#### Experiment 1: feeding behavior after lPBN activation in different physiological contexts

To establish an effective intraperitoneal (i.p.) dose of CNO that is sufficient to reduce feeding behavior, rats expressing DREADDs in lPBN astrocytes were injected with saline or CNO (3, 5, 10 mg/kg i.p.; *n* = 8–9 male rats per dose). No feeding effects were observed at 3 mg, while the 5- and 10-mg/kg injected rats showed a clear anorexic response. Thus, based on this preliminary study, a dose of 5 mg/kg (lowest effective) was selected for further testing. To test the ability of lPBN astrocyte activation to suppress fasting-induced feeding, male and female rats were injected with saline (*n* = 8–12) or CNO (*n* = 12–18) in DREADD+ rats or CNO (DREADD− for CNO control, *n* = 10–12) after overnight fasting. To test whether lPBN astrocyte activation suppresses feeding during the rats’ natural feeding period—the dark cycle, saline or CNO was injected into female (*n* = 6) or male (*n* = 8–9) rats immediately before the dark cycle onset, and the amount eaten was measured 1, 3, and 12 h after injection.

#### Experiment 2: modulation of gut–brain hormone signals by lPBN astrocytes

To determine whether lPBN astrocyte activation interacts with gastrointestinal hormone feeding responses, A) a subthreshold dose (0.1 µg) of CNO was applied directly and unilaterally to the lPBN along with an effective dose of ghrelin (1.0 µg, volume 0.3 µl) in *ad-libitum*-fed (to minimize the potential influence of endogenous ghrelin) male rats during the light cycle (*n* = 9–13) or B) a subthreshold dose (0.1 µg) of CNO was applied directly and unilaterally to the lPBN along with an effective dose of the GLP-1R agonist exendin-4 (0.2 µg, volume 0.3µl) in overnight-fasted (to minimize the potential influence of endogenous GLP-1) male rats (*n* = 10, Latin square design). In A), food intake was measured at 1, 3, and 5 h after injection, and body weight was measured at 24 h. In B), food intake was measured at 1, 3, and 24 h after injection, and body weight was measured at 24 h.

#### Experiment 3: necessity of glutamatergic signaling in lPBN astrocyte-induced feeding suppression

In order to determine whether glutamatergic signaling at the NMDA receptor is necessary to mediate food intake suppression caused by astrocyte activation, the NR2B antagonist Ro-25 (0.15 µg/volume 0.3 µl), or vehicle, was injected at a rate of 1 µl/min, into the lPBN, along with CNO, or saline, delivered at the dose of 5 mg/kg i.p. (1 ml/kg; *n* = 8–10). Food intake was measured at 1 and 5 h, and body weight was measured at 24 h.

#### Experiment 4: effects of lPBN astrocyte activation on food intake after a high-fat and high-sugar diet challenge

In order to determine whether lPBN astrocytes remain effective at suppressing feeding under a high-fat high-sugar diet challenge, rats had *ad-libitum* access to a choice diet of lard and sugar treat and regular chow for 3 weeks. DREADD+ male (*n* = 10–20) and female (*n* = 12–24) rats were then injected with 5 mg/kg of CNO or saline. Food intake was measured at 1, 3, and 12 h, and body weight was also measured at 12 h.

#### Experiment 5: modulation of gut–brain hormone signals by lPBN astrocytes under diet-induced obesity challenge

To determine whether lPBN astrocyte activation interacts with gastrointestinal hormone feeding responses under a high-fat high-sugar diet challenge, A) a subthreshold dose (0.1 µg) of CNO was applied directly and unilaterally to lPBN along with an effective dose of ghrelin (1.0 µg) in *ad-libitum*-fed male rats during the light cycle (*n* = 9, Latin square design) or B) a subthreshold dose (0.1 µg) of CNO was applied directly and unilaterally to lPBN along with an effective dose of a GLP-1R agonist exendin-4 (0.2 µg) in overnight-fasted fed male rats (*n* = 10, Latin square design). In A), food intake was measured at 1, 3, and 5 h after injection, and body weight was measured at 24 h. In B), food intake was measured at 1, 3, and 24 h after injection, and body weight was measured at 24 h.

### Pica test

Pica response was measured as a proxy of emesis in a species not capable of the emetic response. Rats were exposed to kaolin for at least 3 days before the CNO and aCSF control injections in order to familiarize them with the substance before the testing day. Kaolin and chow intake were measured at 1, 3, and 24 h after CNO or control injections in rats food-deprived overnight.

### Perfusion and brain collection

The peritoneal cavity and diaphragm were carefully opened, and the heart was exposed. A cannula attached to a perfusion pump (Watson 120S, Watson-Marlow Fluid Technology Group, Wilmington, MA, USA) was inserted into the heart near the apex and an incision was made into the left ventricle. Cold saline solution was circulated for 3–4 min followed by ice-cold, filtered, 4% paraformaldehyde (PFA)-phosphate-buffered saline (PBS) solution. Brains were isolated and incubated overnight in a 15% sucrose, 4% PFA-PBS solution. They were then transferred to a 30% sucrose-PBS solution until saturation. Samples were frozen on CO_2_-ice prior to brain sectioning with a Leica 3050S cryostat (Leica Biosystems Nussloch GmbH, Nussloch, Germany). Twenty-micrometer coronal slices were collected on SuperFrost+ sample glasses (Thermo Scientific, Waltham, MA, USA) and stored at −80°C.

### Confirmation of DREADD expression in glial cells

RNAscope Multiplex Fluorescent kit (Newark, CA, United States: ACDbio) was utilized to confirm DREADD expression with glial (*Gfap*) mRNA probe. Brain sections were processed using RNAscope for fixed frozen sections using the following protocol provided by the manufacturer: first, brain sections (20 µm) from perfused brains were air-dried for 30 min. Target retrieval was performed in which brain sections were incubated at 99°C in a Retrieval Solution for 7.5 min (Newark, CA, United States: ACDbio), washed 3–5 times with Milli-Q water, and dehydrated for 15 s in 100% ethanol. Protease 3 (Newark, CA, United States: ACDbio) was applied for 1 h at 40°C in the HybEZ oven. The protease was washed off with Milli-Q 3–5 times. The target probe for *Gfap* (Rn-GFAP 407881) and the negative control probes (320871) were applied directly on the sections to cover them completely and incubated at 40°C for 2 h in the HybEZ oven. Next, we applied preamplifier and amplifier probes (AMP1, 40°C for 30 min; AMP2, 40°C for 15 min; AMP3, 40°C for 30 min; AMP4-Alt A for 15 min). Finally, brain sections were incubated for 30 s with DAPI and mounting medium for fluorescence (VECTASHIELD, USA). Fluorescent images of the lPBN were captured using a ×10 and ×40 water immersion magnification lens with an LSM700 Zeiss confocal microscope and processed using ZEN lite software.

### Statistical analysis

All the data are presented as mean ± SEM. Statistical significance was analyzed using Student’s *t*-test for the comparison of two groups or one- or two-way ANOVA with *post-hoc* Sidak’s tests when appropriate (San Diego, CA, United States: GraphPad Software, Inc.). A *P*-value <0.05 was considered statistically significant. Data were plotted using GraphPad Prism (GraphPad Software, Inc., San Diego, CA, USA).

## Results

### Feeding behavior suppression after lPBN activation in different physiological contexts

**Figure 1:**
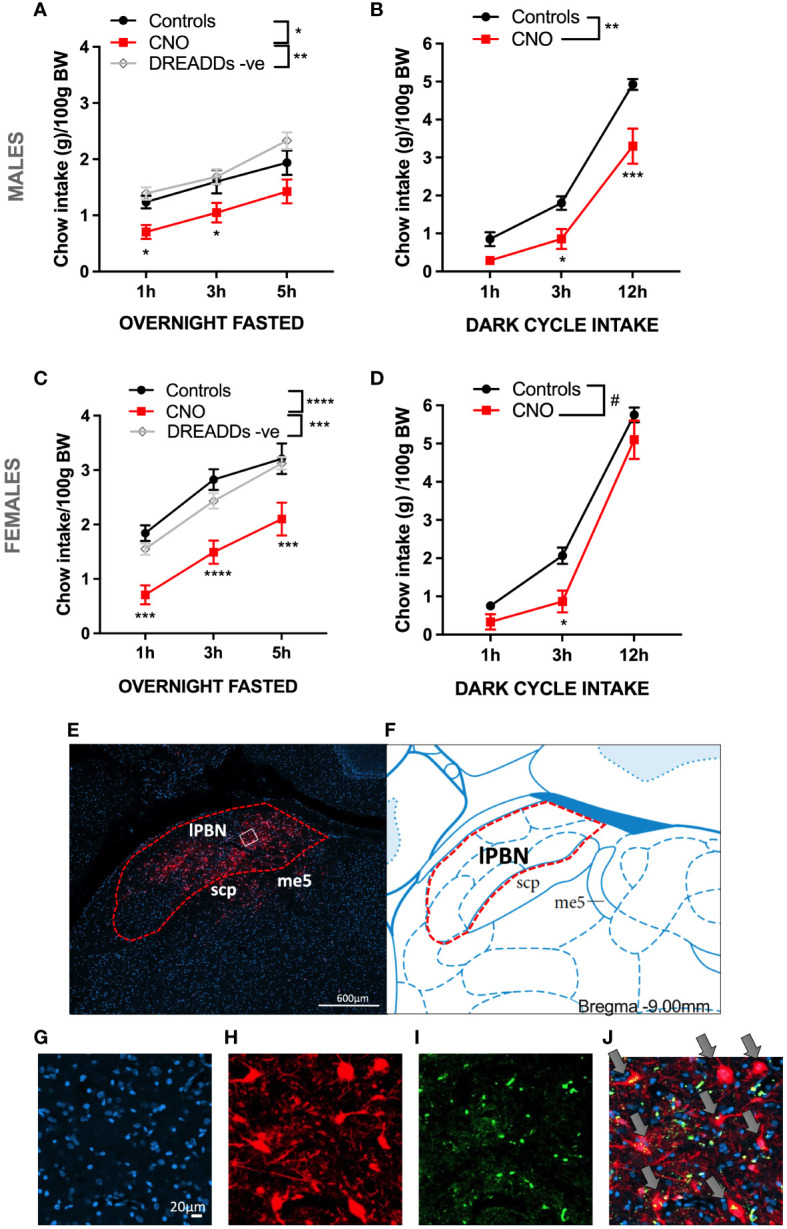
Activation of lateral parabrachial nucleus (lPBN) astrocytes leads to hypophagia in male and female rats. Activation of lPBN astrocytes by IP CNO injection in male rats expressing activational DREADD receptors on lPBN astrocytes leads to reduced chow intake in overnight-fasted rats **(A)**. Activation of lPBN astrocytes also reduces chow intake during the natural feeding period, dark cycle, in *ad-libitum*-fed male rats **(B)**. Similarly, a robust food intake reduction results from lPBN astrocyte activation in overnight-fasted female rats **(C)**. Activation of lPBN astrocytes in female rats during the dark cycle also leads to lower chow intake **(D)**. mCherry expression introduced by the DREADD-carrying AAV indicates that the virus spread throughout the lPBN but not into the medial PBN **(E, F)**. Expression of AAV-introduced label was present exclusively on astrocytes as indicated by the complete overlap of cell bodies expressing *Gfap*, as measured by RNAscope (green), and AAV-introduced mCherry (red) **(G–J)**. DAPI, a nuclear stain is labeled in blue. Fasted study females: *n* = 12 per group, males: *n* = 8–19. Dark cycle study: females: *n* = 6, males: *n* = 8–9. Data are expressed as mean ± SEM. *Post-hoc* tests under line graphs refer to Controls to CNO comparisons (all DREADD positive). #*p* = 0.05, *p < 0.05, **p < 0.01, ***p < 0.001, ****p < 0.0001. DREADD -ve: CNO control rats, DREADD-negative rats.

Chemogenetic activation of lPBN astrocytes (CNO 5 mg/kg i.p.) resulted in feeding behavior suppression in overnight-fasted male rats [two-way ANOVA treatment × time interaction: *F* (4, 68) = 0.8163, *P* = 0.5192; effect of treatment: *F* (2, 34) = 6.033, *P* = 0.0057; effect of time: *F* (2, 68) = 52.33, *P* < 0.0001] (**Figure 1A**). Food intake was expressed as grams eaten per 100 g of body weight as the results were obtained from two batches of rats with significantly different body weights. Similarly, chemogenetic activation of lPBN astrocytes immediately before the dark cycle resulted in feeding suppression in *ad-libitum*-fed male rats [two-way ANOVA chemogenetic activation × time interaction: *F* (2, 30) = 5.00, *P* = 0.0133; effect of chemogenetic activation: *F* (1, 15) = 11.24, *P* = 0.0044; effect of time: *F* (2, 30) = 240.1, *P* < 0.0001]. The onset of anorexia was somewhat delayed compared with that recorded in fasted-refed rats, and significant feeding suppression was detected at 3 and 12 h post-CNO injection (**Figure 1B**). Chemogenetic activation of lPBN astrocytes resulted in a potent feeding behavior suppression also in overnight-fasted female rats [two-way ANOVA treatment × time interaction: *F* (4, 66) = 0.6498, *P* = 0.6290; effect of treatment: *F* (2, 33) = 13.13, *P* < 0.0001; effect of time: *F* (2, 66) = 128.2, *P* < 0.0001] at all time points measured (**Figure 1C**). Also, the dark cycle feeding was significantly suppressed by lPBN astrocyte activation in *ad-libitum*-fed females [two-way ANOVA chemogenetic activation × time interaction: *F* (2, 20) = 3.053, *P* = 0.07; effect of chemogenetic activation: *F* (1, 10) = 4.779, *P* = 0.05; effect of time: *F* (2, 20) = 508.0, *P* < 0.0001]. However, *post-hoc* analysis indicated significant suppression only at the 3-h measurement time point (**Figure 1D**). Data have also been analyzed with the two control groups combined as there were no significant differences in feeding behavior between the DREADD− CNO controls and saline-injected DREADD+ rats. These results are shown in **Supplementary Figure S1**. Analysis of AAV-introduced label from the rats in this study indicated that injections precisely covered the lateral but not medial PBN (**Figures 1E, F**). mCherry fluorescence was visualized directly, without immunohistochemistry enhancement. Moreover, expression of AAV-introduced label was present exclusively on astrocytes as indicated by the complete overlap of cell bodies expressing *Gfap* and mCherry (**Figures 1G–J**), which is consistent with what was previously reported by others using AAV-delivered GFAP promoter (10, 16, 25–27). Anorexia induced by lPBN astrocyte activation was not associated with malaise, as the rats did not consume more kaolin compared with control rats at any of the time points measured (**Supplementary Figure S2**).

### Orexigenic effects of ghrelin are attenuated by lPBN astrocytic activation

**Figure 2:**
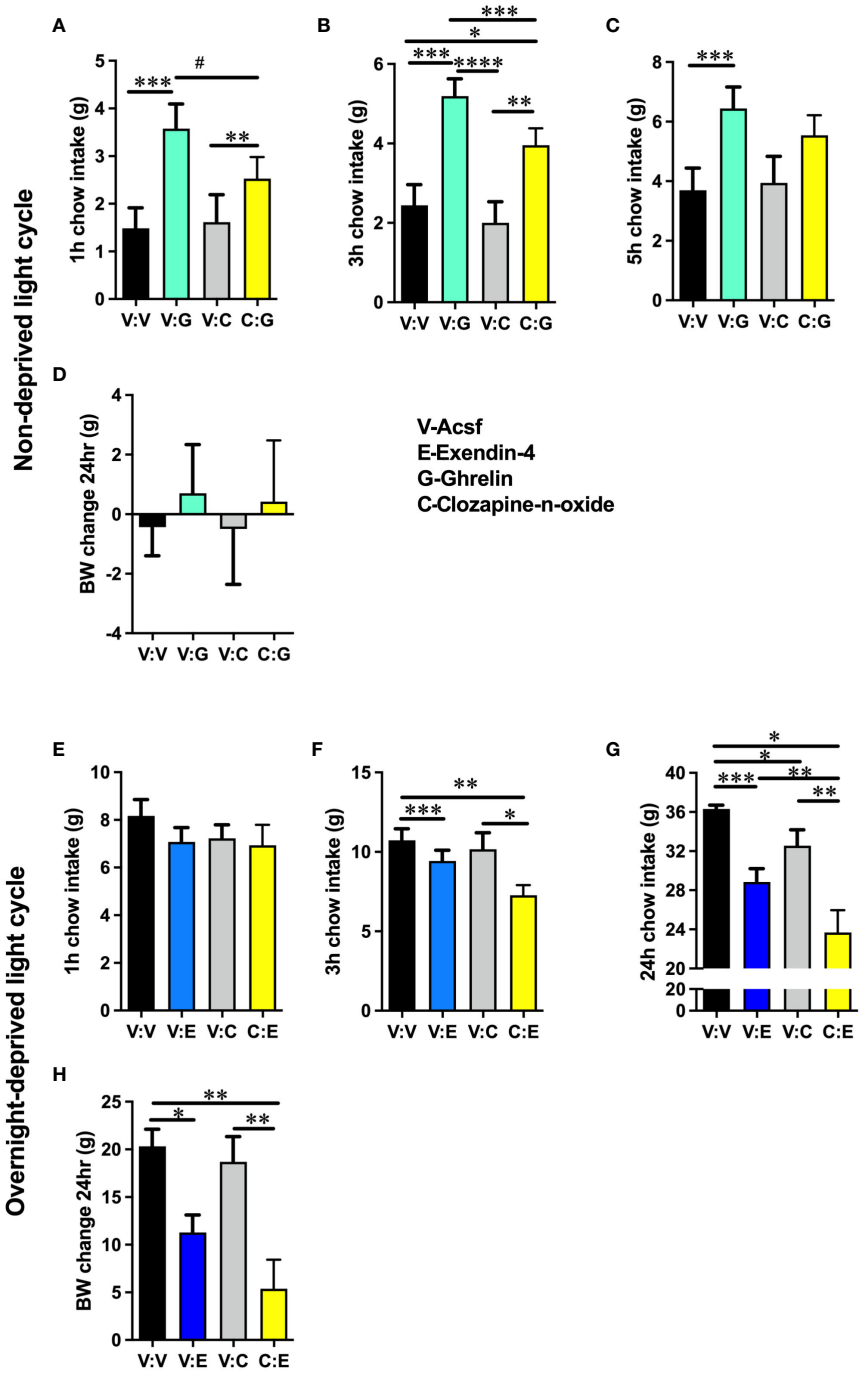
The orexigenic effects of ghrelin are attenuated, and the anorexic effects of exendin-4 are potentiated by lPBN astrocytic activation. Activation of GHSRs in lPBN results in a potent orexigenic response, which is attenuated by chemogenetic activation of lPBN astrocytes in *ad-libitum*-fed male rats at 1 h **(A)** and 3 h **(B)** post injections. At 5 h post-lPBN CNO and ghrelin injections, ghrelin remains orexigenic, but astrocyte-induced attenuation is no longer significant **(C)**. Body weight was not altered by any treatment at the 24-h measurement point **(D)**. At 1 h post-lPBN exendin-4 and CNO injection in male rats, none of the treatments alter chow intake **(E)**. Activation of GLP-1R in lPBN by lPBN-directed exendin-4 infusion results in an anorexic response, which is potentiated by chemogenetic activation of lPBN astrocytes in overnight-fasted male rats at 3 and 24 h time points **(F, G)**. Body weight was reduced by exendin-4 at the 24-h measurement point, but the effect was not potentiated by chemogenetic lPBN astrocytic activation **(H)**. *n* = 9–13. Data are expressed as mean ± SEM. #*p* < 0.1, *p < 0.05, **p < 0.01, ***p < 0.001, ****p < 0.0001.

Activation of growth hormone secretagogue receptor (GHSR) in lPBN resulted in a potent orexigenic response, which was attenuated by chemogenetic activation of lPBN astrocytes in *ad-libitum*-fed male rats [two-way ANOVA at 1 h: interaction: *F* (1, 11) = 5.392, *P* = 0.0404; effect of chemogenetic activation: *F* (1, 11) = 1.053, *P* = 0.3268; effect of ghrelin: *F* (1, 11) = 22.03, *P* = 0.0007; at 3 h: interaction *F* (1, 11) = 2.083, *P* = 0.1768; effect of chemogenetic activation: *F* (1, 11) = 3.682, *P* = 0.0813; effect of ghrelin: *F* (1, 11) = 31.77, *P* = 0.0002; at 5 h: interaction: *F* (1, 8) = 0.8969, *P* = 0.3713; effect of chemogenetic activation: *F* (1, 8) = 0.3382, *P* = 0.5769; effect of ghrelin: *F* (1, 8) = 27.08, *P* = 0.0008] (**Figures 2A–C**). Body weight was not altered by any treatment at the 24-h measurement point (**Figure 2D**).

### Anorexic effects of exendin-4 are potentiated by lPBN astrocytic activation

Activation of GLP-1R in lPBN by lPBN exendin-4 infusion resulted in an anorexic response, which was potentiated by chemogenetic activation of lPBN astrocytes in overnight-fasted male rats at the 3-h time point [two-way ANOVA at 1 h: interaction: *F* (1, 9) = 0.8498, *P* = 0.3807; effect of chemogenetic activation: *F* (1, 9) = 0.6057, *P* = 0.4564; effect of exendin-4: *F* (1, 9) = 1.415, *P* = 0.2647; at 3 h: interaction *F* (1, 9) = 2.306, *P* = 0.1632; effect of chemogenetic activation: *F* (1, 9) = 3.656, *P* = 0.09; effect of exendin-4: *F* (1, 9) = 2.306, *P* = 0.1632; at 24 h: interaction: *F* (1, 9) = 0.7122, *P* = 0.4206; effect of chemogenetic activation: *F* (1, 9) = 8.742, *P* = 0.0160; effect of ghrelin: *F* (1, 9) = 43.84, *P* < 0.0001] (**Figures 2E–G**). Body weight was reduced by exendin-4 at the 24-h measurement point, but the effect was not potentiated by chemogenetic lPBN astrocytic activation (**Figure 2H**).

### Glutamatergic signaling is necessary for lPBN astrocyte-induced feeding suppression

**Figure 3:**
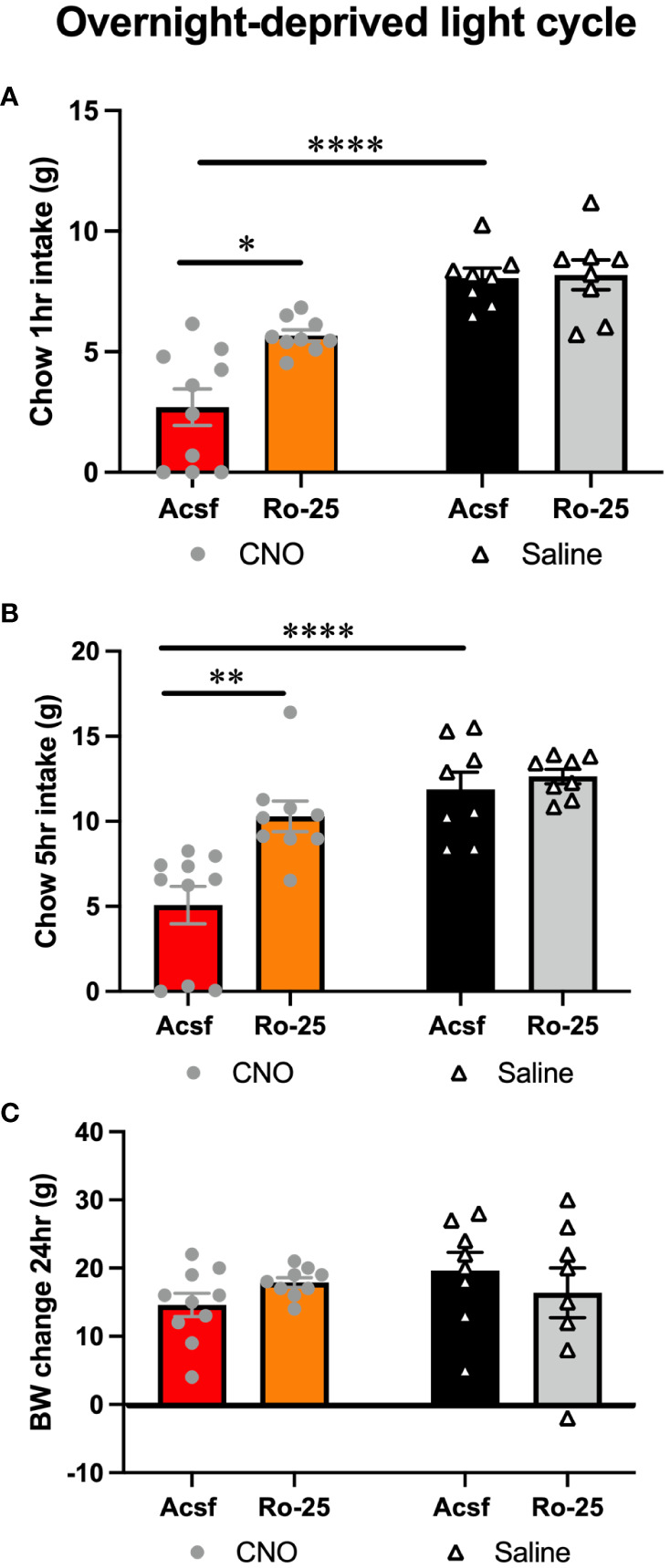
Glutamatergic signaling is necessary for lPBN astrocyte-induced feeding suppression. Feeding suppression induced by lPBN astrocyte activation is attenuated by pharmacological blockade of NMDA receptors in male rats at 1 **(A)** and 5 h post-lPBN injection **(B)**. Body weight was not altered by any treatment 24 h after injection **(C)**. *n* = 8–10 Data are expressed as mean ± SEM. *p < 0.05, **p < 0.01, ****p < 0.0001.

Feeding suppression induced by lPBN astrocyte activation was attenuated by pharmacological blockade of NMDA receptors in male rats [two-way ANOVA at 1 h: interaction: *F* (1, 4) = 6.573, *P* = 0.06; effect of chemogenetic activation: *F* (1, 9) = 53.80, *P* < 0.0001; effect of Ro-25: *F* (1, 9) = 7.917, *P* = 0.02; at 5 h: interaction *F* (1, 31) = 5.693, *P* = 0.02; effect of chemogenetic activation: *F* (1, 31) = 24.01, *P* < 0.0001; effect of Ro-25: *F* (1, 31) = 10.30, *P* = 0.003] (**Figures 3A, B**). Body weight was not altered by any treatment 5 h after injection, although it is unlikely for meaningful changes to adipose tissue or muscle to be detected at such a short time interval from treatment (**Figure 3C**).

### LPBN astrocyte anorexia under high-fat high-sugar diet challenge

**Figure 4:**
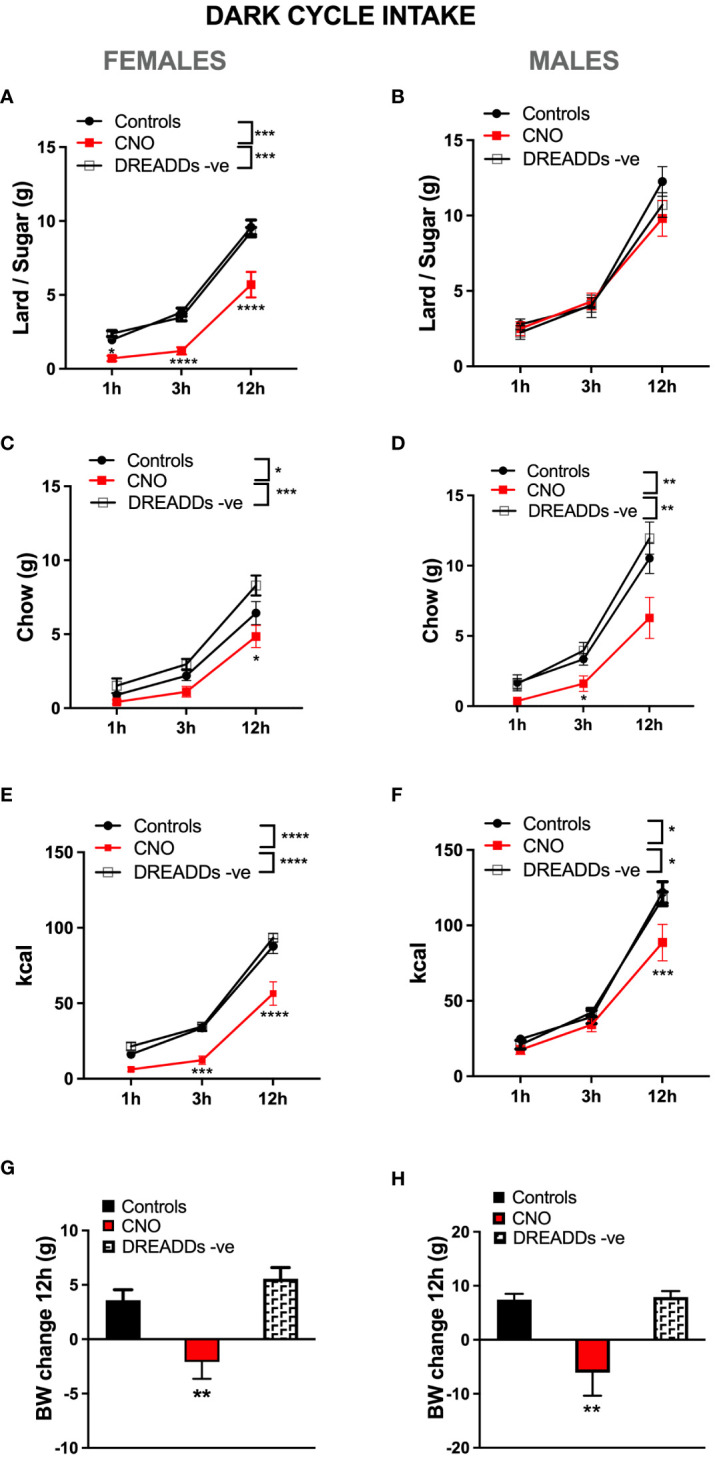
Sex-divergent effects of lPBN astrocyte activation-induced anorexia under high-fat high-sugar diet challenge. In females challenged by a choice of high-fat and high-sugar diet and chow, lPBN astrocytic activation robustly reduced palatable lard–sugar mix intake at all time points measured **(A)**. Females in this diet context also significantly, albeit less robustly, reduced their chow intake, significant only at 12 h post-injection **(B)**. Given the significant reductions in both sources of calories, also total caloric intake was reduced in female rats after lPBN astrocyte activation at all time points measured **(C)**. Astrocyte activation also led to body weight gain suppression in females **(D)**. In contrast to the results obtained in females, males challenged by a choice of high-fat high-sugar diet and chow did not reduce their palatable food intake at any time points measured in response to lPBN astrocytic activation **(E)**. However, males did reduce their chow intake similar to females **(F)**. Total caloric intake was also reduced in male rats after lPBN astrocyte activation, albeit less robustly than in females and only at 12 h post-injection **(G)**. Astrocyte activation resulted in significant body weight gain suppression also in males **(H)**. Females: *n* = 12 per group, males: *n* = 10 per group. *Post-hoc* tests under the line graphs refer to Controls to CNO comparisons (all DREADD-positive). Data are expressed as mean ± SEM. *p < 0.05, **p < 0.01, ***p < 0.001, ****p < 0.0001. DREADD -ve: CNO control rats, DREADD-negative rats injected with CNO.

In females challenged by a choice of high-fat high sugar diet and chow, lPBN astrocytic activation robustly reduced palatable food intake at all time points measured [two-way ANOVA treatment × time interaction: *F* (4, 66) = 4.460, *P* = 0.003; effect of treatment: *F* (2, 33) = 25.31, *P* < 0.0001; effect of time: *F* (2, 66) = 337.5, *P* < 0.0001; **Figure 4A**]. Females in this diet context also significantly, albeit less robustly, reduced their chow intake [two-way ANOVA treatment × time interaction: *F* (4, 66) = 2.083, *P* = 0.09; effect of treatment: *F* (2, 33) = 8.065, *P* = 0.0014; effect of time: *F* (2, 66) = 147.2, *P* < 0.0001; **Figure 4B**]. Given significant reductions in both sources of calories, also total caloric intake was reduced in female rats after lPBN astrocyte activation [two-way ANOVA treatment × time interaction: *F* (4, 66) = 4.494, *P* = 0.0028; effect of treatment: *F* (2, 33) = 23.78, *P* < 0.0001; effect of time: *F* (2, 66) = 389.1, *P* < 0.0001; **Figure 4C**]. Astrocyte activation also resulted in significant body weight gain suppression in females [treatment effect: *F* (2, 33) = 10.83; *P* = 0.0002; **Figure 4D**]. In contrast to the results obtained in females, males challenged by a choice of high-fat high-sugar diet and chow did not reduce their palatable food intake at any time points measured in response to lPBN astrocytic activation [two-way ANOVA treatment × time interaction: *F* (4, 54) = 1.694, *P* = 0.1648; effect of treatment: *F* (2, 27) = 0.6033, *P* < 0.0001; effect of time: *F* (2, 40) = 188.3, *P* < 0.0001; **Figure 4E**]. However, males did reduce their chow intake similar to females [two-way ANOVA treatment × time interaction: *F* (4, 54) = 3.247, *P* = 0.0185; effect of time: *F* (2, 36) = 135.3, *P* < 0.0001; effect of treatment: *F* (2, 27) = 7.153, *P* = 0.0032; **Figure 4F**]. Total caloric intake was also reduced in male rats after lPBN astrocyte activation, albeit less robustly than in females [two-way ANOVA treatment × time interaction: *F* (4, 54) = 3.086, *P* = 0.0232; effect of time: *F* (2, 54) = 271.8, *P* = 0.0001; effect of treatment: *F* (2, 27) = 4.386, *P* = 0.02; **Figure 4G**]. Astrocyte activation resulted in significant body weight gain suppression also in males [*F* (2, 27) = 9.201; *P* < 0.0009; **Figure 4H**]. For the above data, the two control groups (DREADD and CNO control groups) have been presented and analyzed separately; however, since as hypothesized, there were no significant differences in feeding behavior between the DREADD− CNO controls and saline-injected DREADD+ rats, we also analyzed and presented the results with the two control groups collapsed, and these data are shown in **Supplementary Figure S3**.

### Modulation of anorexia induced with GLP-1R activation by lPBN astrocytes under diet-induced obesity challenge

**Figure 5:**
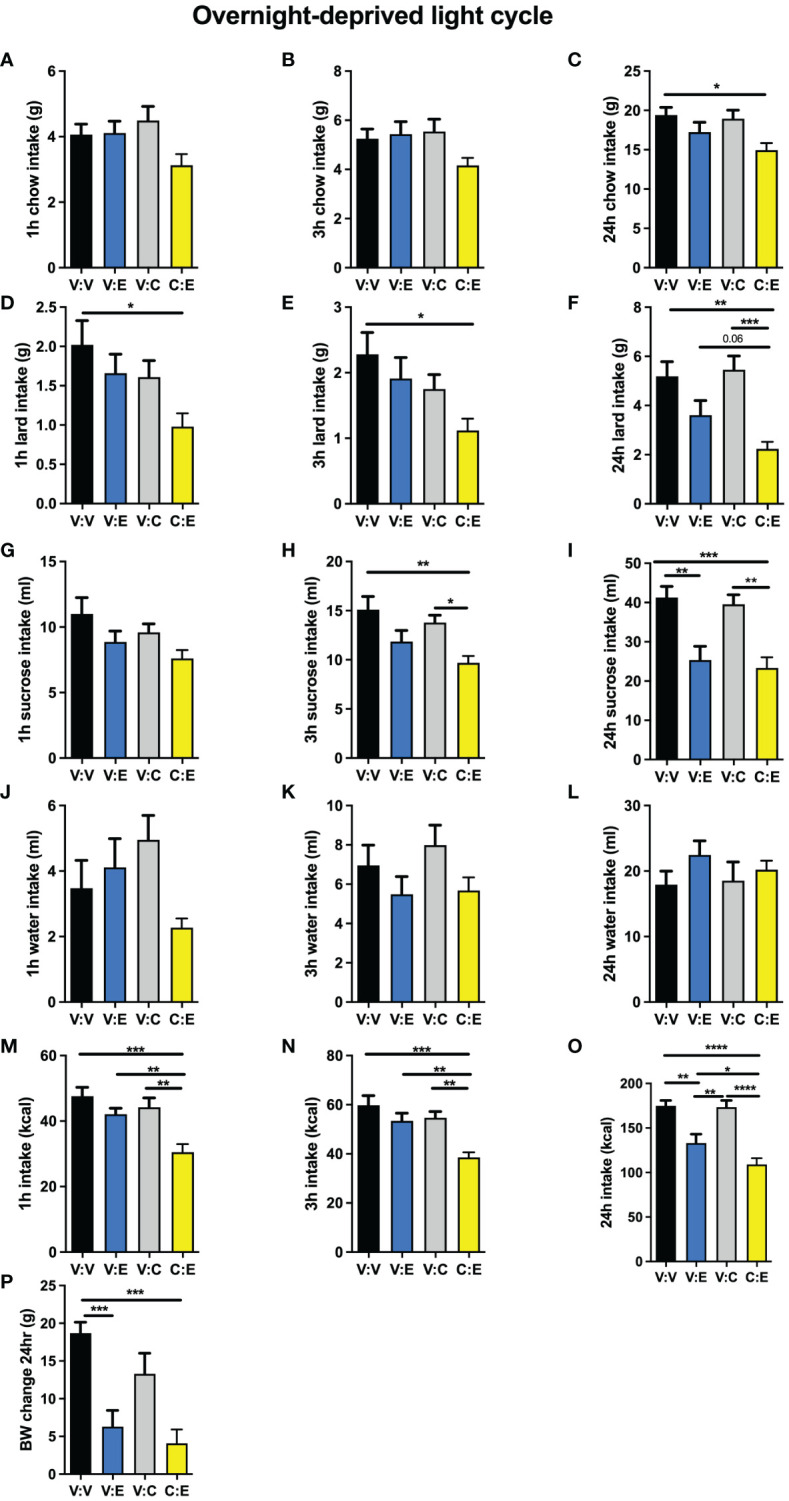
Modulation of anorexia induced with GLP-1R activation by lPBN astrocytes under diet-induced obesity challenge. In rats challenged by a high-fat high-sugar choice diet, GLP-1R activation in the lPBN by exendin-4 was not effective at reducing chow intake and neither was the deliberately selected subthreshold dose of CNO at 1 h **(A)**, 3 h **(B)**, or 24 h **(C)** post-injection. However, at the 24-h time point, rats treated with both exendin-4 and CNO significantly reduced their chow intake, suggesting a synergistic effect of the two treatments **(C)**. Exendin-4 alone was also not effective at reducing lard intake at any time points measured **(D–F)**; however, at 24 h, concomitant activation of lPBN astrocytes via CNO injection and GLP-1R with exendin-4 resulted in a potent suppression of lard intake to less than 40% of the amount consumed by control rats **(F)**. Sucrose intake was not altered by any of the treatments at 1 h **(G)**; at 3 h, only the combination of exendin-4 and CNO resulted in reduced intake **(H)**. At 24 h, both exendin-4 alone and its combination with CNO resulted in a comparable suppression of sucrose intake **(I)**. Chow, 30% sucrose solution, and lard were provided separately in order to distinguish the preference for each. None of the treatments affected water intake at any time points measured **(J–L)**. Total caloric intake was reduced only by the combined application of exendin-4 and CNO at 1 and 3 h **(M, N)**, while at 24 h, both exendin-4 alone and the combination reduced intake although the combination was still significantly more effective than GLP-1R activation alone **(O)**. Body weight was also reduced by exendin-4 alone and the combination of CNO and exendin-4 to a similar extent **(P)**. Males: *n* = 10. V, Vehicle; E, Exendin-4; C, CNO. Data are expressed as mean ± SEM. #*p* < 0.1, *p < 0.05, **p < 0.01, ***p < 0.001, ****p < 0.0001.

In male rats challenged by a high-fat high-sugar choice diet, GLP-1R activation in the lPBN by exendin-4 alone was not effective at reducing chow intake at any time points measured (**Figures 5A–C**). This is in contrast to the potent effect obtained by the same treatment in chow-fed rats. CNO injection alone, at a deliberately selected subthreshold dose, was also not effective at reducing chow intake. However, at the 24-h time point, rats treated with both exendin-4 and CNO significantly reduced their chow intake, suggesting a synergistic effect of the two treatments (**Figure 5C**). Exendin-4 alone was also not effective at reducing lard intake at any time points measured (**Figures 5D–F**); however, at 24 h, concomitant activation of lPBN astrocytes via CNO injection and GLP-1R with exendin-4 resulted in a potent suppression of lard intake to less than 40% of the amount consumed by control rats (**Figure 5F**). Sucrose intake was not altered by any of the treatments at 1 h (**Figure 5G**); at 3 h, only the combination of exendin-4 and CNO resulted in reduced intake (**Figure 5H**). At 24 h, both exendin-4 alone and its combination with CNO resulted in a comparable suppression of sucrose intake (**Figure 5I**). None of the treatments affected water intake (**Figures 5J–L**). Overall, total caloric intake was reduced only by the combined application of exendin-4 and CNO at 1 and 3 h (**Figures 5M, N**), while at 24 h, both exendin-4 alone and the combination reduced intake, although the combination was still significantly more effective than GLP-1R activation alone (**Figure 5O**). Body weight was also reduced by exendin-4 alone by the combination of CNO and exendin-4 to a similar extent (**Figure 5P**).

### High-fat high-sugar maintenance alters the interaction of ghrelin and lPBN astrocytic activation on feeding behavior

**Figure 6:**
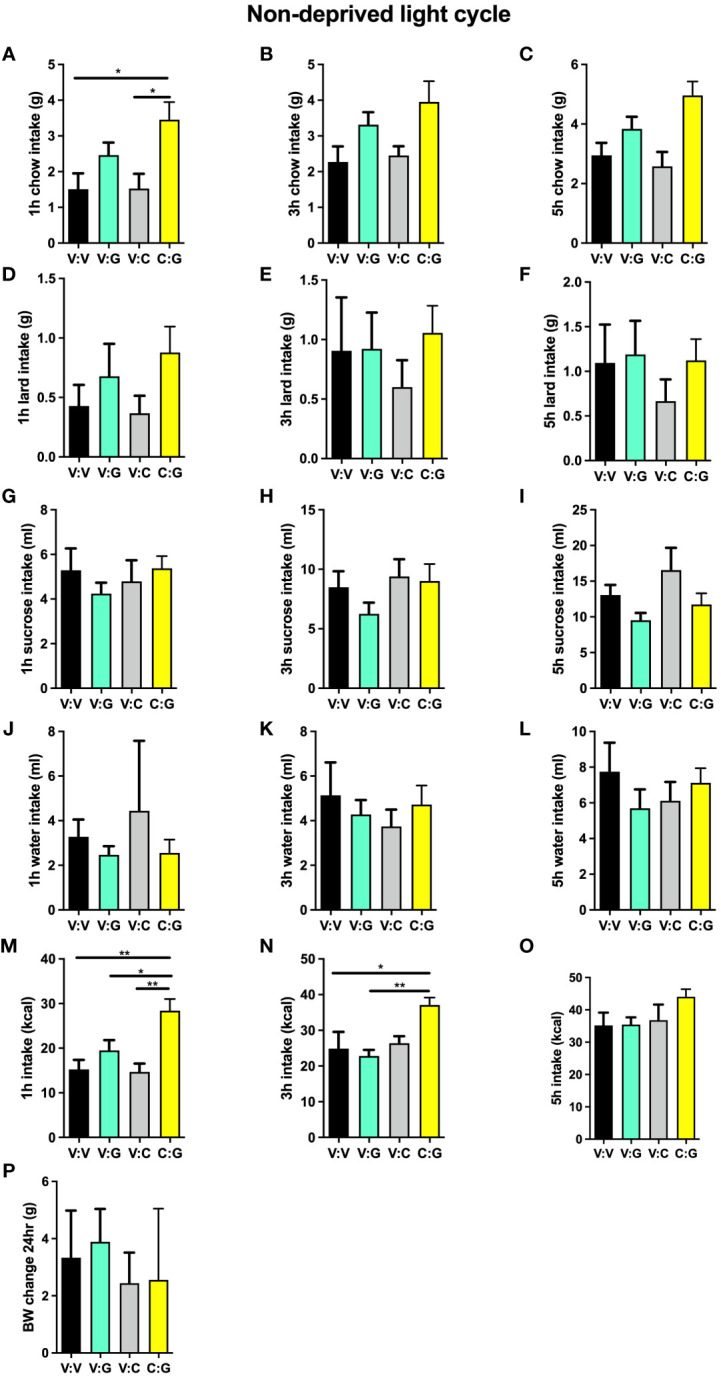
High-fat high-sugar maintenance alters the interaction of ghrelin and lPBN astrocytic activation on feeding behavior. Under obesogenic diet maintenance, ghrelin lPBN infusions alone did not affect chow intake **(A–C)**, lard intake **(D–F)**, sucrose intake **(G–I)**, or total caloric intake **(M–O)** at any of the time points measured. Surprisingly, however, and in contrast to the attenuating effect of lPBN astrocyte activation on ghrelin’s hyperphagia in chow-maintained rats, lPBN astrocyte activation enhanced ghrelin hyperphagia at 1 and 3 h post-injections **(M, N)**. Chow, 30% sucrose solution, and lard were provided separately in order to distinguish the preference for each. Water intake **(J–L)** and body weight **(P)** were not affected by any of the treatments. *n* = 9. V, Vehicle; G, Ghrelin; C, CNO. Data are expressed as mean ± SEM. *p < 0.05, **p < 0.01.

Under obesogenic diet maintenance, activation of lPBN GHSR alone was no longer orexigenic, in line with the literature indicating ghrelin resistance in obese subjects. Thus, ghrelin lPBN infusions alone did not affect chow intake (**Figures 6A–C**), lard intake (**Figures 6D–F**), sucrose intake (**Figures 6G–I**), or total caloric intake (**Figures 6M–O**) at any of the time points measured. Surprisingly, however, and in contrast to the attenuating effect of lPBN astrocyte activation on ghrelin’s hyperphagia in chow-maintained rats, lPBN astrocyte activation enhanced ghrelin hyperphagia at 1 and 3 h post-injections (**Figures 6M, N**). Water intake (**Figures 6J–L**) and body weight (**Figure 6P**) were not affected by any of the treatments.

## Discussion

The control of feeding behavior is not restricted to the hypothalamus or the NTS; however, to date, very little is known about astrocytic contribution to feeding behavior control outside of these brain regions. Our data strongly support the idea that astrocytes in the lPBN control feeding behavior. Activation of lPBN astrocytes produced a robust feeding suppression under natural feeding conditions, as well as after a fasting/refeeding challenge, in both male and female rats. The anorexic effect of lPBN astrocytic activation persisted under high-fat high-sugar diet challenge, reducing chow intake in both sexes; however, astrocytic activation reduced palatable food intake only in female rats. Glutamatergic signaling in the lPBN was necessary for lPBN astrocytes to produce anorexia. Moreover, lPBN astrocyte activation interacted with stomach and intestinal hormone signals but did so in a partly divergent manner when rats were maintained on a high-fat high-sugar compared with a standard diet.

Even though astrocytes are the most abundant glial cell type in the mammalian CNS (28), their role in energy balance regulation, or food intake control more specifically, remains poorly understood. In fact, very little is known about any functional role of lPBN astrocytes. A recent study, however, suggests that activation of lPBN astrocytes increases wakefulness (29). Here, we found that chemogenetic activation of lPBN astrocytes effectively suppressed feeding behavior in rats during the natural feeding period (dark cycle) of the rat, with a 3-h onset. Given prior data on the ability of lPBN astrocytes to modulate wakefulness, one worry could be that rats may choose to rest rather than eat after chemogenetic astrocyte activation. However, activation of astrocytes increased rather than decreased wakefulness; thus, increased resting is unlikely the reason for reduced feeding behavior.

Challenges to energy homeostasis in the form of fasting reorganize arcuate nucleus astrocytes (30); thus, it is possible that astrocytes are also acutely responsive and interact with fasting in other brain areas known to change their activity in response to fasting or fasting-related signals (17, 18, 20, 22, 31). Here, we found that even under conditions of an overnight (dark cycle) fast, activation of lPBN astrocytes remains effective at inducing feeding suppression. In fact, the suppression of feeding after fasting appeared to be greater and with faster onset than that achieved during the dark cycle.

Astrocytes can influence synaptic activity by the release of glutamate, but they also participate in other key aspects of glutamate processing in the CNS including uptake of released glutamate and *de-novo* synthesis of glutamate from glucose; the latter two activities are likely exclusive to astrocytes (11, 32, 33). As discussed above, metabolic state can alter astrocytic ability to take up glutamate. Here, we found that intact glutamate signaling is required for the anorexic effect of lPBN astrocytic activation. Given that we used a pharmacological approach to block glutamate signaling, it is not possible to distinguish whether the glutamate required for this effect originates from the activated astrocyte or neurons, released as a result of neuronal activation downstream of the affected astrocytes. Future studies evaluating selective disruption of glutamate release of neurons or astrocytes may determine which of those mechanisms is relevant here. Our results regarding the necessity of glutamate are also in line with the previously discovered complete feeding suppression effect of glutamate signaling in the lPBN, engaged by disinhibition of this nucleus, by suppression of the hypothalamic inhibitory input to the lPBN (23, 34).

Despite greater understanding among preclinical scientists of sex differences in CNS energy balance control and a nudge on the part of major funding bodies to include females in preclinical research, majority of the studies investigating astrocytic contribution to feeding control evaluated exclusively male rats or did not clearly report the sex of their subjects. Thus, the overall idea of astrocytic control of feeding behavior in females remains largely unknown. However, astrocytic morphology in the hypothalamus is sex divergent, from birth through adulthood (35, 36). Astrocytes are also directly responsive to sex steroids, as they express both estrogen and androgen receptors (37–39). Furthermore, the levels of the astroglia marker GFAP are sex divergent, at least in the hypothalamus, and this divergence is gonadal steroid-dependent (35). Sex differences in the responses of astrocytes to obesity in the hypothalamus have been suggested as one of the factors contributing to fewer detrimental effects of obesogenic diet in females (40). Still, sex differences, or female astrocytes overall, have not been investigated in the lPBN. Here, we show that chemogenetic activation of lPBN astrocytes in females results in anorexia. While in lean rats both males and females reduced their chow intake in response to astrocytic activation, in diet-induced obese rats, we discovered that astrocytic activation is highly effective in females in suppressing both chow and palatable food intake, yet in males, it is only effective at reducing chow.

Astrocytes in different brain regions may be differentially affected by diet-induced obesity (41). For example, while rats challenged with an obesogenic diet had reduced numbers of astrocytes (but not microglia) in the dorsal vagal complex, the numbers of astrocytes in the arcuate nucleus of the hypothalamus remained unaltered (42). In the current study, rats maintained on an obesogenic diet for at least 3 weeks retained their ability to reduce feeding behavior after lPBN astrocyte activation. In females, this activation was effective at suppressing the intake of both a palatable high caloric food and also chow. In males, however, astrocytic activation was not able to suppress palatable diet intake. We previously found that male and female rats respond differently to diet-induced obesity, where female rats are more protected and resistant to the metabolic dysfunction of diet-induced obesity (43). The sex difference in the ability of lPBN astrocytes to suppress palatable food intake may be one of the CNS mechanisms contributing to female resistance to diet-induced obesity. However, despite a much less robust feeding suppression, males lost more body weight compared with female rats, suggesting potential energy expenditure effects of this treatment. We have previously shown the thermogenic effects of various nutritional status signals acting on the lPBN in males (22, 24, 44); thus, it is feasible that astrocytic activation may also result in increased thermogenesis. Future studies will be needed to confirm this idea.

Astrocytes in the hypothalamus and the dorsal vagal complex can detect nutrients and directly respond to adipokines like leptin and other well-established feeding signals (42, 45–48). Here, we hypothesized that also lPBN astrocytes can interact with signals conveying nutrient status like GLP-1, released after a meal, or ghrelin, released from the stomach during fasting. The role of GLP-1 and its receptor in the CNS in energy balance control is well-established and exploited therapeutics in type 2 diabetes and obesity (49–51). We and others previously found that GLP-1 acts at the level of the lPBN to reduce food intake and body weight (17, 20). Here, we found a synergistic effect of a subthreshold dose of CNO and an effective dose of exendin-4, a clinically utilized GLP-1R agonist, on chow intake. The synergistic effect of GLP-1R and astrocytic activation shown here may have resulted from a direct interaction of these signals in astrocytes, given that in other brain areas, for example the NTS, GLP-1R are expressed directly on the astrocytes (14, 52–54). Activation of GLP-1R on NTS astrocytes results in increased cAMP levels in astrocytes, and pharmacological inhibition of astrocytes prevents GLP-1R/exendin-4-induced anorexia (14), demonstrating a key role for astrocytes in GLP-1-induced feeding suppression. It is also possible that these signals interact indirectly by parallel activation of astrocytes and neuronal GLP-1R, with the anorexic signal converging on downstream target cells in the lPBN or other brain areas. Moreover, these two possibilities are not mutually exclusive.

Ghrelin is mainly secreted by the stomach and its acylated form promotes food intake primarily through its action on the GHSR receptor in the CNS. Its levels are high during fasting or before meals. Ghrelin's metabolic actions are partly mediated through modulation of hypothalamic astrocytes (55). Ghrelin also affects hypothalamic astrocytes by depolarizing them, likely indirectly via GHSRs on neighboring neurons (30). Furthermore, GHSR activation increases glial coverage of hypothalamic AgRP neurons which morphologically eliminates gating inhibitory synapses on these neurons. Ghrelin also increases glutamate uptake and reduces glucose uptake by hypothalamic astrocytes, which may be protective for neighboring neurons during energetic challenges like fasting (55). This may also be one of the mechanisms via which ghrelin and its mimetics can protect against glutamate excitotoxicity (56). These actions are likely mediated by the direct action of ghrelin on GHSR present in astrocytes (55, 57). Here, we found that activation of GHSR in lPBN results in a potent orexigenic response, which is attenuated by chemogenetic activation of lPBN astrocytes in *ad-libitum* chow-fed male rats. Surprisingly, however, and in contrast to the attenuating effect of lPBN astrocyte activation on ghrelin’s hyperphagia in chow-maintained rats, lPBN astrocyte activation enhanced ghrelin hyperphagia. While the reduced response to ghrelin in the lPBN, or ghrelin resistance, we found under the obesogenic diet challenge is potentially a beneficial adaptive response to ensuing obesity, the switch in astrocytic contribution to ghrelin’s response from attenuation to potentiation is likely maladaptive neuroregulatory response to obesity. The altered interaction of astrocytes with feeding signals in animals maintained on an obesogenic diet found here is in line with previous data. For example, in the DVC, while astrocytic activity is partly necessary for the anorexic effects of leptin in lean rats, astrocytes become dispensable for leptin’s feeding suppression in obese rats (42). Moreover, in rats susceptible to diet-induced obesity, glia-mediated synaptic reorganization favors orexigenic signaling (58).

Collectively, current findings uncover a novel role for lPBN astrocytes in feeding behavior control. This role is supported by the interaction with well-established feeding signals and is sex divergent under obesogenic palatable diet conditions and requires intact lPBN glutamatergic signaling.

## Data availability statement

The original contributions presented in the study are included in the article/**Supplementary Material**. Further inquiries can be directed to the corresponding author.

## Ethics statement

The animal study was approved by University of Gothenburg Animal Ethics Committee. The study was conducted in accordance with the local legislation and institutional requirements.

## Author contributions

DM: Conceptualization, Data curation, Formal analysis, Writing – original draft, Writing – review & editing. JR: Writing – original draft, Writing – review & editing, Data curation. IM: Writing – original draft, Writing – review & editing, Data curation. OS: Writing – original draft, Writing – review & editing, Data curation. SB: Writing – original draft, Writing – review & editing, Data curation. KE: Writing – original draft, Writing – review & editing, Data curation. J-PK: Writing – original draft, Writing – review & editing, Data curation. KS: Conceptualization, Funding acquisition, Resources, Supervision, Visualization, Writing – original draft, Writing – review & editing.

## Supplementary material

Supplementary Figure S1 Anorexic effect of activation of lPBN astrocytes in chow-fed rats (with control groups combined). Activation of lPBN astrocytes by IP CNO injection in male and female rats expressing activational DREADD receptors on lPBN astrocytes leads to reduced chow intake at 1, 3, and 5h post injection, in overnight-fasted rats. Females: n = 12–24, males: n= 18–19. Data are expressed as mean ± SEM. ***p* < 0.01, *****p* < 0.0001.

Supplementary Figure S2 Activation of lPBN astrocytes is not associated with nausea as measured by the PICA response. The saline-injected DREADD positive rats were used as controls. N=12. Data are expressed as mean ± SEM

Supplementary Figure S3 Anorexic effect of activation of lPBN astrocytes in high-fat high-sugar diet fed rats (with control groups combined). In females challenged by a choice of high-fat and high-sugar diet and chow, lPBN astrocytic activation robustly reduced palatable lard-sugar mix intake at all time points measured **(A)**. Females in this diet context also significantly, albeit less robustly, reduced their chow intake **(B)**. Given significant reductions in both sources of calories, also total caloric intake was reduced in female rats after lPBN astrocyte activation at all time points measured **(C)**. Astrocyte activation also led to body weight gain suppression in females **(D)**. In contrast to the results obtained in females, males challenged by a choice of high-fat high sugar diet and chow, did not reduce their palatable food intake at any time points measured in response to lPBN astrocytic activation **(E)**. However, males did reduce their chow intake similarly to females **(F)**. Total caloric intake was also reduced in male rats after lPBN astrocyte activation, albeit less robustly than in females and only at 12h post injection **(G)**. Astrocyte activation resulted in significant body weight gain suppression also in males (H). Females: n = 12, males: n=10–20. Data are expressed as mean ± SEM. **p* < 0.05, ***p* < 0.01, ****p* < 0.001, *****p* < 0.0001.
