# Supplementary figures and images for: Lateral parabrachial nucleus astrocytes control food intake
Source: Front Endocrinol (Lausanne). 2024 Jun 3;15:1389589. doi: 10.3389/fendo.2024.1389589 (PMC11180714; doi:10.3389/fendo.2024.1389589)

MALES

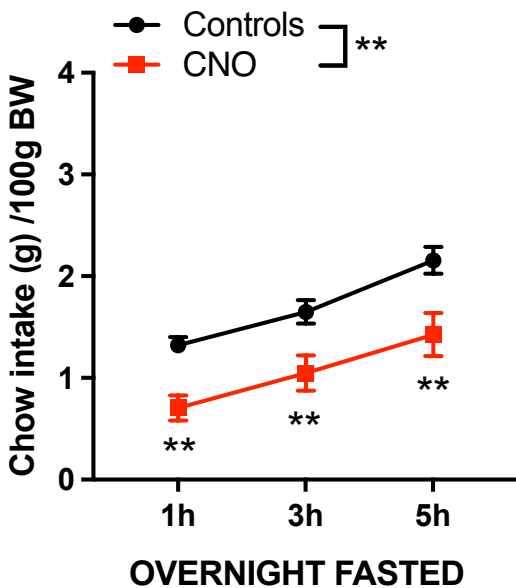

FEMALES

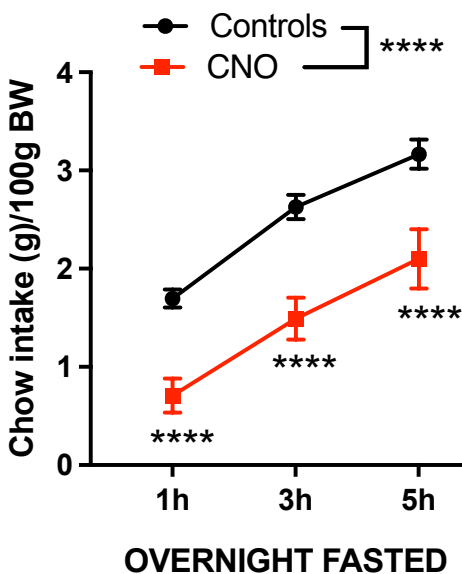

Figure S1

Supplement: Supplementary Figure S1 — Anorexic effect of activation of lPBN astrocytes in chow-fed rats (with control groups combined). Activation of lPBN astrocytes by IP CNO injection in male and female rats expressing activational DREADD receptors on lPBN astrocytes leads to reduced chow intake at 1, 3, and 5h post injection, in overnight-fasted rats. Females: n = 12–24, males: n= 18–19. Data are expressed as mean ± SEM. **p < 0.01, ****p < 0.0001. [file DataSheet_1.pdf]

Figure S2

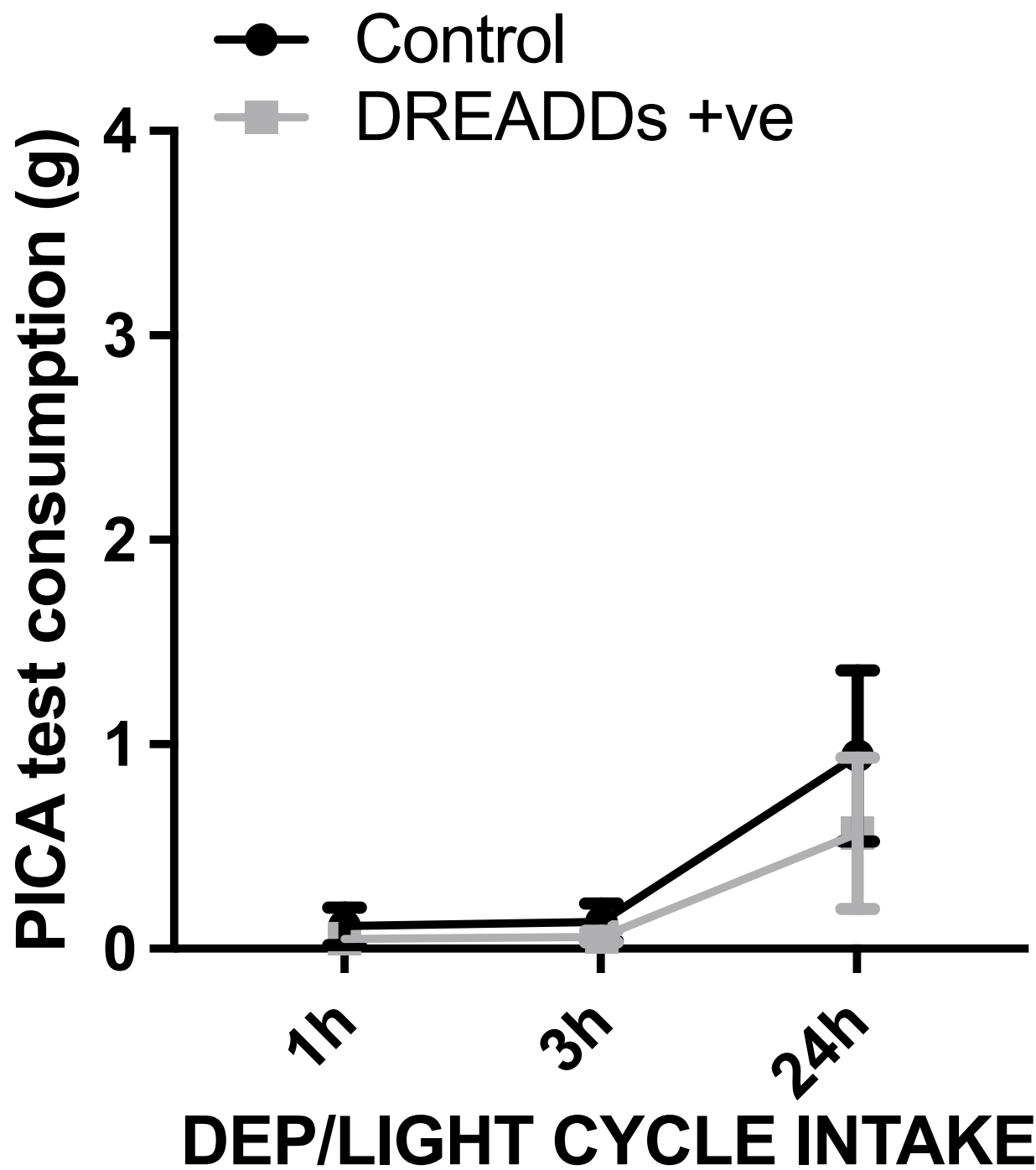

Supplement: Supplementary Figure S2 — Activation of lPBN astrocytes is not associated with nausea as measured by the PICA response. The saline-injected DREADD positive rats were used as controls. N=12. Data are expressed as mean ± SEM [file DataSheet_2.pdf]

Figure S3

DARK CYCLE INTAKE

FEMALES

MALES

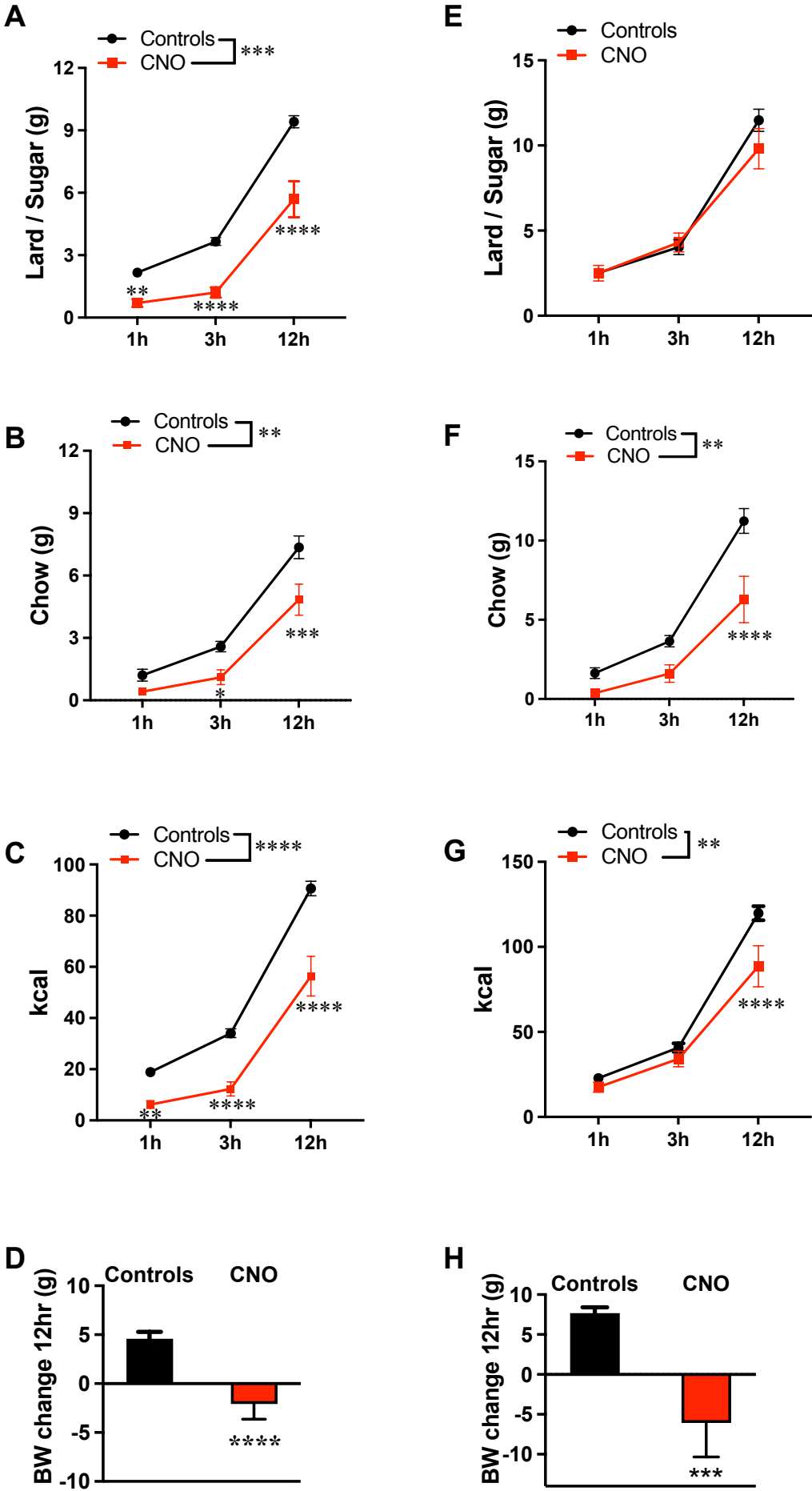

Supplement: Supplementary Figure S3 — Anorexic effect of activation of lPBN astrocytes in high-fat high-sugar diet fed rats (with control groups combined). In females challenged by a choice of high-fat and high-sugar diet and chow, lPBN astrocytic activation robustly reduced palatable lard-sugar mix intake at all time points measured (A). Females in this diet context also significantly, albeit less robustly, reduced their chow intake (B). Given significant reductions in both sources of calories, also total caloric intake was reduced in female rats after lPBN astrocyte activation at all time points measured (C). Astrocyte activation also led to body weight gain suppression in females (D). In contrast to the results obtained in females, males challenged by a choice of high-fat high sugar diet and chow, did not reduce their palatable food intake at any time points measured in response to lPBN astrocytic activation (E). However, males did reduce their chow intake similarly to females (F). Total caloric intake was also reduced in male rats after lPBN astrocyte activation, albeit less robustly than in females and only at 12h post injection (G). Astrocyte activation resulted in significant body weight gain suppression also in males (H). Females: n = 12, males: n=10–20. Data are expressed as mean ± SEM. *p < 0.05, **p < 0.01, ***p < 0.001, ****p < 0.0001. [file DataSheet_3.pdf]
